# Spatial activity participation in childhood and adolescence: consistency and relations to spatial thinking in adolescence

**DOI:** 10.1186/s41235-020-00239-0

**Published:** 2020-09-16

**Authors:** Emily Grossnickle Peterson, Adam B. Weinberger, David H. Uttal, Bob Kolvoord, Adam E. Green

**Affiliations:** 1 School of Education, American University, Washington, DC 20016 USA; 2 Department of Psychology, Georgetown University, Washington, DC USA; 3 Department of Psychology, Northwestern University, Evanston, IL USA; 4 College of Integrated Science and Engineering, James Madison University, Harrisonburg, VA USA; 5 Department of Psychology and Interdisciplinary Program in Neuroscience, Georgetown University, Washington, DC USA; 6 Penn Center for Neuroaesthetics, University of Pennsylvania, Philadelphia, PA USA

**Keywords:** Spatial activities, Spatial cognition, Spatial skills, Sex differences

## Abstract

**Background:**

Prior research has revealed positive effects of spatial activity participation (e.g., playing with blocks, sports) on current and future spatial skills. However, research has not examined the degree to which spatial activity participation remains stable over time, and little is known about how participating in spatial activities at multiple points in development impacts spatial thinking. In this study, adolescents completed measures of spatial thinking and questionnaires assessing their current and previous participation in spatial activities.

**Results:**

Participation in childhood spatial activities predicted adolescent spatial activity participation, and the relation was stronger for females than for males. Adolescents’ current participation in spatial activities predicted spatial thinking skills, whereas participation in childhood spatial activities predicted adolescents’ spatial habits of mind, even when accounting for factors such as gender and academic performance. No cumulative benefit was incurred due to participating in spatial activities in both childhood and adolescence, and a lack of spatial activities in childhood was not made up for by later spatial activity participation.

**Conclusions:**

These findings reveal a consistently positive relationship in spatial activity participation between childhood and adolescence. Results highlight the importance of participating in spatial activities during childhood, and underscore the differential impact that participation in spatial activities during childhood versus adolescence has on different facets of adolescents’ spatial thinking. Implications for the timing of interventions is discussed.

## Significance

For teachers and researchers interested in supporting Science, Technology, Engineering, and Mathematics (STEM) education, the concern that students’ spatial thinking is often underdeveloped is met with encouraging evidence that spatial skills are malleable in response to training and intervention (Uttal et al., 2013). In addition to laboratory and classroom-based training paradigms, participation in everyday activities that require spatial thinking (i.e., spatial activities) predicts both concurrent and future spatial thinking. To broaden understanding of how interventions may be optimized to increase spatial thinking, we recruited a large sample of high school students to complete spatial thinking assessments and questionnaires to assess their engagement with spatially-based activities. We investigated (a) the degree to which participation in spatial activities shows consistency over time, and (b) whether the association between spatial activity participation and spatial thinking differs by when the participation occurred (i.e., during childhood vs. adolescence). Gender differences related to these two primary questions were also explored. We observed consistency in spatial activity participation; individuals who played with spatial toys as children participated more in spatial activities (e.g., basketball, dance) as adolescents. This relationship was particularly strong for female participants. Associations between spatial activities and spatial thinking differed based on when spatial activity participation occurred, and the measure of spatial thinking.

## Background

### Spatial thinking skills and strategies

Spatial thinking is a multifaceted construct, including skills and strategies involved in imagining objects from different angles, visually searching a scene, or picturing transformations of 2-D and 3-D objects (NRC, 2006; Uttal et al., 2013). Students’ success in spatial tasks (i.e., their spatial skills) are important for learning in STEM domains and reliably predict student achievement in STEM (Wai, Lubinski, & Benbow, 2009). In addition to spatial skills, the tendency to approach problems in a spatial way—referred to as spatial strategies or spatial habits of mind—is associated with learning in STEM (DeMers & Vincent, 2007; Kim, 2011; Kim & Bednarz, 2013). Indeed, developing spatial problem solving strategies is an important outcome for science education, above and beyond the development of spatial skills (NRC, 2006). In the present study, we examine the development of these two facets of spatial thinking (i.e., spatial skills and spatial habits of mind) in relation to participation in spatial activities.

### Development of spatial thinking

Spatial skills are malleable and develop through dedicated practice, schooling, and activity experiences (Uttal et al., 2013). Because spatial skills improve with training and experience (Uttal et al., 2013), understanding the degree to which spatial skills vary as a function of spatial activity participation is important for improving spatial thinking (Levine, Ratliff, Huttenlocher, & Cannon, 2012; Quaiser-Pohl & Lehmann, 2002; Vander Heyden, Huizinga, & Jolles, 2017). Broadly defined, spatial activities are activities that involve reasoning about qualities of space (e.g., distance, proportion), practicing mental visualization (e.g., imagining spatial layouts or spatial trajectories), and observing the positions of physical objects. These activities can include sports, play activities, artistic endeavors, and technological pursuits.

**Fig. 1:**
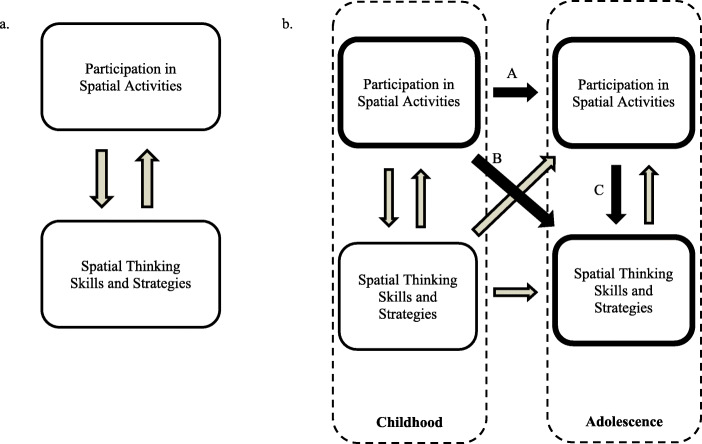
Theoretical relations between spatial activities and spatial thinking. These relations are bi-directional in nature and can be examined at a single point in time (**1a**) or across development (**1b)**. The bolded boxes and arrows represent the constructs and relations examined in the present study

Participating in spatial activities is positively associated with spatial thinking (Feng, Spence, & Pratt, 2007; Gittler & Gluck, 1998; Pietsch & Jansen, 2012; Nazareth, Herrera, & Pruden, 2013; Terlecki, Newcombe, & Little, 2008; see Dearing et al., 2012 for an exception). As depicted in Fig. 1a, this relation is considered to be bi-directional: participation in spatial activities may improve spatial thinking skills and strategies, and individuals with a greater capacity for spatial thinking may seek out opportunities to engage in spatial activities. Baenninger and Newcombe’s (1989) meta-analysis of the relation between spatial activities and spatial ability in adults revealed this correlation to be modest.

Additional studies in the 30 years since the publication of this meta-analysis have also identified a positive association between spatial activity participation and spatial thinking skills in childhood and adolescence (see Fig. 1b; e.g., Levine et al., 2012; Jirout & Newcombe, 2015; Moreau, Clerc, Mansy-Dannay, & Guerrien, 2012; see Dearing et al., 2012 for an exception). Childhood spatial toys, such as blocks and jigsaw puzzles, provide concrete experience observing, arranging, and discussing the physical location of objects in space (Borriello & Liben, 2018; Verdine et al., 2019). Participating in puzzle and block play has been shown to be positively associated with spatial skills (Jirout & Newcombe, 2015; Levine et al., 2012). Moreover, children participating in block building and spatial play interventions showed benefits above and beyond improvements in active control groups, and provide evidence of a causal relation (Casey et al., 2008; Vander Heyden et al., 2017). Similarly, activities such as building replicas (e.g., model trains), artistic endeavors (e.g., drawing, knitting, needlework), and mechanical activities (e.g., car repair) involve working with representations in two and three dimensions and also promote spatial skill development (Doyle, Voyer, & Cherney, 2012; Newcombe, Bandura, & Taylor, 1983).

Spatial thinking may also be supported by participation in sports such as soccer and basketball, as well as movement-related activities such as dance (Pietsch & Jansen, 2012; Voyer, Nolan, & Voyer, 2000; Weckbacher & Okamoto, 2012). These activities involve reasoning about static and dynamic positioning (Jansen, Ellinger, & Lehmann, 2018; Moreau et al., 2012). A quasi-experimental study found that adolescents taking extended physical education classes performed better on a mental rotation test than adolescents in regular physical education classes (Jansen et al., 2018). This finding is in line with correlational evidence that individuals who regularly practiced sports have better spatial skills than those who do not (Moè, Jansen, & Pietsch, 2018; Pietsch & Jansen, 2012; see Quaiser-Pohl & Lehmann, 2002 for an exception). Not all sports, however, are spatial in nature. For example, training in wrestling improved mental rotation task performance more than training in running (Moreau et al., 2012). Spatial skills are also associated with activities such as playing music (Pietsch & Jansen, 2012), technical activities (Quaiser-Pohl & Lehmann, 2002), and computer experience (Terlecki & Newcombe, 2005).

### Spatial activities across development

People can participate in spatial activities across the lifespan, including throughout childhood and adolescence. Assessing with the consistency of spatial activity participation across development (Fig. 1b path A) is an important point of inquiry for educators and policy advocates interested in fostering spatial thinking capabilities. Understanding how and when children and adolescents participate in spatial activities will help us design better activities that are tailored to specific ages. Despite this clear potential for advancement, little is known about the degree to which spatial activity participation is consistent over time. That is, do individuals who participate in spatial activities in childhood continue participating in more spatial activities in adolescence, or do activity preferences change?

We are aware of only two studies that assessed participation in spatial activity participation at multiple points in time (Moè et al., 2018; Robert & Héroux, 2004); however, the focus of these studies was the relationship between activity participation and spatial skills rather than consistency in participation over time. Nonetheless, studies of participation in specific domains of spatial thinking (e.g., sports, blocks, videogames) provide some insight on how activity preferences may evolve over time. Participation in sports during childhood and adulthood are modestly correlated (Perkins, Jacobs, Barber, & Eccles, 2004; Richards, Williams, Poulton, & Reeder, 2007; Telama et al., 2005). Video game use in early adulthood is correlated with videogame play in childhood but displays a curvilinear relationship, with increased use beginning in childhood and peaking in adolescence (Ream, Elliot, & Dunlap, 2013). Research on the selection of gendered activities also suggests a high level of consistency in activity selection across childhood and adolescence, with boys and girls consistently favoring toys that match their own gender (Todd et al., 2017). Thus, while the existing literature points to relative consistency in specific domains of spatial activity participation (i.e., sports, videogames), additional work focused on general patterns of spatial activities is needed. Differences in general activity preferences by gender suggest that such a relationship may function differently for boys and girls, especially in light of the predominance of masculine-gendered spatial activities (Doyle et al., 2012; Lauer, Udelson, Jeon, & Lourenco, 2015).

### Gender differences in spatial activities

Gender differences in spatial activity participation is one possible explanation for why gender differences in spatial thinking tend to favor males. There are two general mechanisms that may explain this effect. First, gender differences in spatial thinking may result from gender differences in spatial activity participation (i.e., mean-level differences in spatial activities result in mean-level differences in spatial thinking skills and strategies). Second, gender differences in spatial thinking may result from gender differences in the strength of the relation between spatial activities and spatial thinking (i.e., gender moderates the relation between spatial activities and spatial thinking skills and strategies).

With regard to mean-level differences, males tend to engage more frequently with activities and toys that contain spatial elements than females do (Baenninger & Newcombe, 1995; Cherney & Voyer, 2010; Nazareth et al., 2013; Signorella, Krupa, Jamison, & Lyons, 1986). Gender differences in play and toy preferences emerge in infancy, and male-stereotyped toys tend to be more spatial in nature (Doyle et al., 2012; Lauer et al., 2015). Gendered stereotypes of toys have held over recent decades, and are reflected in the content of children’s rooms (MacPhee & Prendergast, 2019). Despite evidence that boys tend to engage in more spatial activities, it is important to note that not all spatial activities fit this gendered pattern. For instance, Levine et al. (2012) found no gender differences in puzzle play during home observations in childhood. Moreover, in the United States, girls’ participation in sports has been increasing since the introduction of Title IX of the 1972 Education Amendments required equal opportunities for sports participation regardless of sex (Stevenson, 2007).

In addition, gender may moderate the effect of spatial activities such that spatial activities differentially benefit males’ and females’ spatial thinking. Some studies have found that females benefit from spatial activities more than males (Feng et al., 2007; Moè et al., 2018; Quaiser-Pohl & Lehmann, 2002). Researchers have suggested that this effect occurs because males may already receive sufficient support for spatial skills through a variety of experiences, whereas females may require additional spatial experiences to catch up to their male peers (Quaiser-Pohl & Lehmann, 2002; Tzuriel & Egozi, 2010). When considering the quality of puzzle play in children, higher quality play was related to the spatial skills of girls but not boys (Levine et al., 2012). Alternatively, findings from other studies indicate that males benefit from spatial activities more than females (Connor & Serbin, 1977; González-Calero, Cózar, Villena, & Merino, In press; Wong & Yeung, 2019). Researchers hypothesize that this may be due to males engaging with spatial activities in a more spatial way (Wong & Yeung, 2019). For instance, Lego® blocks marketed to girls encourage stereotypically feminine traits (e.g., friendship, beauty), whereas, Lego® blocks marketed to boys promoted more active play (e.g., cars, professions; Reich, Black, & Foliaki, 2018).

However, not all findings support the hypothesis that gender moderates the relation between spatial activities and spatial thinking. (e.g., Jansen et al., 2018; Moreau et al., 2012; Vander Heyden et al., 2017). Results from meta-analyses suggest that males and females benefit similarly from spatial training and spatial activities Baenninger & Newcombe, 1989; Uttal et al., 2013). There is also evidence that retrospective reports of childhood activities correlate with adult spatial skills even after controlling for gender (Doyle et al., 2012). Given these conflicting findings, it is therefore important to examine whether gender moderates the relation between spatial activities and spatial thinking as it unfolds in childhood and adolescence.

### Present study

The present study explored three research questions concerning spatial activity participation, and how participation relates to spatial skills and spatial habits of mind. First, Research Question 1 examined the extent to which engagement in spatial activities remains consistent from childhood to adolescence (Fig. 1b path A). That is, does participating in spatial activities as a child predict how frequently an adolescent partakes in spatial activities? Because general activity preferences remain stable (Perkins et al., 2004; Ream et al., 2013; Richards et al., 2007), we anticipated that individuals who participated more frequently in spatial activities during childhood would continue to participate in spatial activities during adolescence.

Research Question 2 examined whether participation in spatial activities at these two developmental stages (i.e., childhood and adolescence) predicts spatial thinking skills and spatial habits of mind (Fig. 1b paths B & C). Individuals who participate in spatial activities tend to score higher on tests of spatial thinking (Doyle et al., 2012; Nazareth et al., 2013), but whether this trend differs based on when in development participation occurs (e.g., childhood or adolescence) is unknown. Thus, we compared the relative impact of participating at different points during development, including possible interactive effects. For instance, it is possible that individuals incur additional benefit from participating in spatial activities in both childhood and adolescence, or that participating in spatial activities during adolescence can make up for a lack of spatial activity participation in childhood. Analyses were conducted separately for spatial skills and spatial habits of mind given that spatial skills and habits of mind are correlated yet distinct facets of spatial thinking (Kim & Bednarz, 2013; NRC, 2006).

Finally, for Research Question 3, we examined whether gender moderated the relations found in Research Questions 1 and 2. In relation to Research Question 1, we investigated whether gender moderated the relation between childhood activities and adolescent activities. General activity participation is most consistent for activities that are gendered, suggesting that stability in spatial activity engagement may vary by gender as well. In relation to Research Question 2, we explored whether the impact of participation in spatial activities during childhood and adolescence on spatial thinking differed by gender. Although debate remains, prior work has provided some evidence that gender may mediate the ameliorating effects of spatial activities on spatial thinking (Levine et al., 2012; Moè et al., 2018; Wong & Yeung, 2019).

## Methods

### Participants

Participants were 346 high school students (59.25% female) in one of six public schools in Northern Virginia, a suburb of Washington, DC. Students were in grades 10–12 (*M*_age_ = 16.61, *SD*_age_ = .57) and were recruited from study hall, science classes, and math classes to take part in a larger study examining the effects of science education on spatial ability. Fifty-one participants (14.74%) identified as Hispanic. Non-Hispanic participants included students who identified as White (*n* = 208; 60.12%), Black (*n* = 17; 4.91%), Native American (*n* = 1; < 1%), and Asian (*n* = 32; 9.25%), with 5.49% of students identifying in multiple racial categories (*n* = 19) and 2.02% of students identifying according to a category not explicitly listed (*n* = 7). Race and ethnicity data were missing for 3 participants. Most participants (*n* = 286; 82.66%) reported that their mother graduated from a 4-year college or university.

### Measures

#### Childhood spatial activities

The Childhood Activities Questionnaire (CAQ) developed by Cherney and Voyer (2010) asks participants to think back to the frequency with which they played with toys or engaged in activities in childhood. In the present study childhood was defined as “before you entered high school.” The 27-items (see Appendix) are broken into subscales including 13 spatial activities (e.g., puzzles, blocks) and 14 non-spatial activities (e.g., stuffed animals, coloring) based on prior validation and factor analytic research (Cherney & Voyer, 2010). For each of the activities participants were asked to rate their frequency of participation during childhood on a 6-point scale from *Never* to *Always.* The summed score on the spatial items was analyzed in the present study (possible range: 13–78; α = .76).

#### Adolescent spatial activities

The short form of the Spatial Activities Questionnaire (SAQ) developed by Signorella et al. (1986) includes 30 spatial activities (see Appendix) divided into three 10-item subscales: Masculine (e.g., tackle football, car repair), Feminine (e.g., gymnastics, knitting multi-color patterns), and Neutral (e.g., ping pong/table tennis, dodgeball). The 30 items on the short form were selected from an initial pool of 118 items based on the strength of the item loadings (Signorella et al., 1986). For each of the activities, participants were instructed to provide a retrospective report to “indicate the degree to which you have participated in each of the following activities during high school.” Ratings were made on a 6-point scale from *Never* to *More than Once a Week* and were summed to form a total score (possible range: 30–180, α = .82).

#### Spatial skills

Spatial skills were measured using the Paper Folding test included as the VZ-2 subscale in the Kit of Factor-Referenced Cognitive Tests (Ekstrom, French, Harman, & Dermen, 1976). The 20-item test is completed in two sections. Three minutes are allocated for each section with a break in between sections. Each item contains 2–4 images depicting the steps of a piece of paper being folded and with holes punched through the entire thickness of the paper after it is folded. Dotted lines are used to represent where the paper has been folded and small circles are used to represent the holes punched through the paper. Participants are asked to imagine where the holes would be if the paper was unfolded and to select which of the five answer choices correctly demonstrated that answer. Consistent with the published instructions, participants were informed that their score on the test would be calculated by subtracting a proportion of the number of items solved incorrectly from the number of items solved correctly, and thus they were encouraged not to guess. The penalty for each incorrect response was 0.2 points. Possible scores ranged from 0 to 20 and test reliability was high (α = .79).

#### Spatial habits of mind inventory

Spatial habits of mind were measured using the Spatial Habits of Mind Inventory (SHOMI) developed by Kim and Bednarz (2013). The measure contains 28 items across five dimensions: pattern recognition (e.g., “I am curious about patterns in information or data, that is, where things are and why they are where they are”), spatial description (e.g., “I use spatial terms such as scale, distribution, pattern, and arrangement”), visualization (e.g., “When I am thinking about a complex idea, I use diagrams, maps, and/or graphics to help me understand”), spatial concept use (e.g., “When trying to solve some types of problems, I tend to consider location and other spatial factors”), and spatial tool use (e.g., “I use maps and atlases, including digital versions, frequently”; Kim & Bednarz, 2013, p.168). Participants reported how much each item reflected their “thoughts, beliefs, or actions” on a 5-point Likert scale from *Strongly Disagree* to *Strongly Agree.* Scores on the SHOMI were summed (α = .88), with possible scores ranging from 28 to 140.

### Procedure

Participants who returned signed parental consent forms could participate in the study, and students signed informed assent forms if they agreed to participate. Participants completed the measures during their study hall period at their high school. Paper Folding was administered first, with the recommended time limits of three minutes for each of the two sections and a brief pause in between the sections. The researcher read aloud the instructions for this task and answered any questions. Then, participants received a packet containing the SHOMI, spatial activities measures, and a demographic survey. Participants were given as much time as needed to complete these measures, and typically took 20–30 min. The Preliminary SAT (PSAT) was given free of charge to students during the fall of each academic year, and scores were obtained from students’ academic records (missing: *n* = 36; 10.40%).

## Results

Descriptive statistics, bivariate correlations, and gender differences for all measures of spatial thinking and spatial activity participation are presented in Table 1. Note that all t-tests for gender differences were performed with an unequal variance; a Kolmogorov-Smirnov test indicated inequality distribution by gender for all variables except Paper Folding (all *p*s < 0.03).

**Table 1:** Bivariate correlations for spatial activities, spatial thinking measures, and covariates

	N	Mean	Male	Female	1	2	3	4	5	6	7	8	9	10
1. CAQ	345	41.73 (9.94)	45.74^a^ (10.29)	38.95 ^a^ (8.70)	1									
2. SAQ-Overall	345	50.84 (12.43)	49.26 (11.17)	51.93 (13.15)	0.39**	1								
3. SAQ-Masculine	345	15.31 (5.33)	17.77 ^b^ (6.30)	13.61 ^b^ (3.70)	0.51**	0.64**	1							
4. SAQ-Feminine	345	14.21 (5.23)	11.30 ^c^ (2.13)	16.21 ^c^ (5.78)	0.09	0.72**	0.11	1						
5. SAQ-Neutral	345	21.36 (6.16)	20.21 (5.55)	22.16 (6.43)	0.27**	0.85**	0.33**	0.52**	1					
6. Paper Folding	341	11.12 (4.20)	11.71 (4.16)	10.72 (4.18)	0.08	0.10	0.07	0.06	0.10	1				
7. SHOMI	346	101.94 (12.54)	104.19 (13.20)	100.40 (11.85)	0.37**	0.20*	0.24**	0.08	0.13	0.21**	1			
8. PSAT	310	159.08 (24.83)	159.87 (24.91)	158.53 (24.83)	0.02	−0.06	−0.12	− 0.04	0.006	0.42**	0.17	1		
9. Gender (1 = male)	346	0.41		–	0.34**	−0.11	0.39**	−0.46**	− 0.16	0.12	0.15	0.03	1	
10. Race (1 = non-white)	346	0.38		–	−0.06	−0.07	− 0.14	− 0.006	− 0.008	−0.13	− 0.11	−0.19*	− 0.005	1
11. Maternal education (1 = completed college)	346	0.83		–	0.05	−0.005	−0.03	− 0.04	0.04	0.17	0.002	0.29**	0.007	−0.27**

*SAQ* Adolescent spatial activities questionnaire, *CAQ* Childhood activities questionnaire, *SHOMI* Spatial Habits of Mind Inventory

*Note*: * *p* ≤ .05; ** *p* ≤ .01 after controlling for family-wise error rates using a Bonferroni correction; Superscript letters indicate a significant mean difference by gender

Spatial habits of mind was correlated with spatial activity participation, both during childhood (CAQ; *r* = 0.37, *p* < 0.001, Bonferonni-corrected) and adolescence (SAQ; *r* = 0.20, *p* = 0.01, Bonferonni-corrected). CAQ scores were positively associated with overall spatial activity participation during adolescence (*r* = 0.39, *p* < 0.001, Bonferonni-corrected), as well as with the masculine (*r* = 0.51, *p* < 0.001, Bonferonni-corrected) and neutral (*r* = 0.27, *p* < 0.001, Bonferonni-corrected) SAQ subscales. CAQ scores were not correlated with feminine adolescent spatial activity participation (*r* = 0.09), although this may be influenced by the CAQ’s disproportionate focus on masculine spatial activities. Consistent with this interpretation, a one-sample t-test indicated significantly higher CAQ scores for male (*M* = 45.74, *SD* = 10.29) than female participants (*M* = 38.95, *SD* = 8.70; t (267.42) = − 6.41, *p* < 0.01, Bonferonni-corrected). Notably, no such gender differences were observed for the overall SAQ. In line with prior work, *t*-tests indicated higher masculine spatial activity participation for male participants (*t* (206.52) = − 7.06, *p* < 0.01, Bonferonni-corrected) and higher feminine spatial activity participation for female participants (*t* (275.13) = 11.10, *p* < 0.01, Bonferonni-corrected). Finally, bivariate correlations identified an association between spatial skills (operationalized with the Paper Folding test) and spatial habits of mind (*r* = 0.21, *p* = 0.003, Bonferonni-corrected) but not between spatial skills and spatial activity participation during childhood (CAQ: *r* = 0.07) or adolescence (SAQ: *r* = 0.10).

### Consistency of spatial activity participation

Bivariate correlations revealed a significant association between CAQ and SAQ scores. To provide a more precise assessment of the relationship between childhood and adolescent activities (Research Question 1), we ran a linear regression predicting adolescent spatial activities (SAQ) from childhood spatial activities (CAQ), along with the following covariates: PSAT score, gender, race/ethnicity (white = 0, non-white = 1), and maternal college completion (1 = graduated from 4-year college). The model accounted for significant portion of the variance in SAQ scores (F_5,302_ = 17.06, adjusted *R*^*2*^ = .21, *p* < .001; Table 2). Results revealed that childhood spatial activities were predictive of later adolescent activities (*b* = .60, *β* = .47, *SE* = .07, *p* < .001).

**Table 2:** Linear regression models predicting adolescent spatial activities

	Model 1 (No interaction)					Model 2 (Interaction term)				
	t	B	β	95% CI	*p*	t	B	β	95% CI	*p*
**SAQ**										
CAQ	8.69	.60	.47	[.46, .73]	**<.001**	7.71	.74	.58	[.55, .93]	**<.001**
Gender	−5.19	−7.14	−.28	[−9.85, −4.43]	**<.001**	.87	5.18	.20	[−6.61, 16.97]	.39
Gender X CAQ	–	–	–	–	–	−2.11	−.29	−.55	[−.56, −.02]	**.04**
Race	− 1.18	− 1.16	−.06	[−4.31, 1.09]	.24	− 1.34	− 1.84	−.07	[− 4.53, .85]	.18
Maternal education	−.02	−.05	−.001	[−3.62, 3.53]	.98	.13	.24	.01	[−3.33, 3.80]	.90
PSAT	−1.56	−.04	−.08	[−.10, .01]	.12	−1.73	−.05	−.09	[−.10, .01]	.08

*SAQ* Adolescent spatial activities questionnaire, *CAQ* Childhood activities questionnaire – spatial subscale

**Fig. 2:**
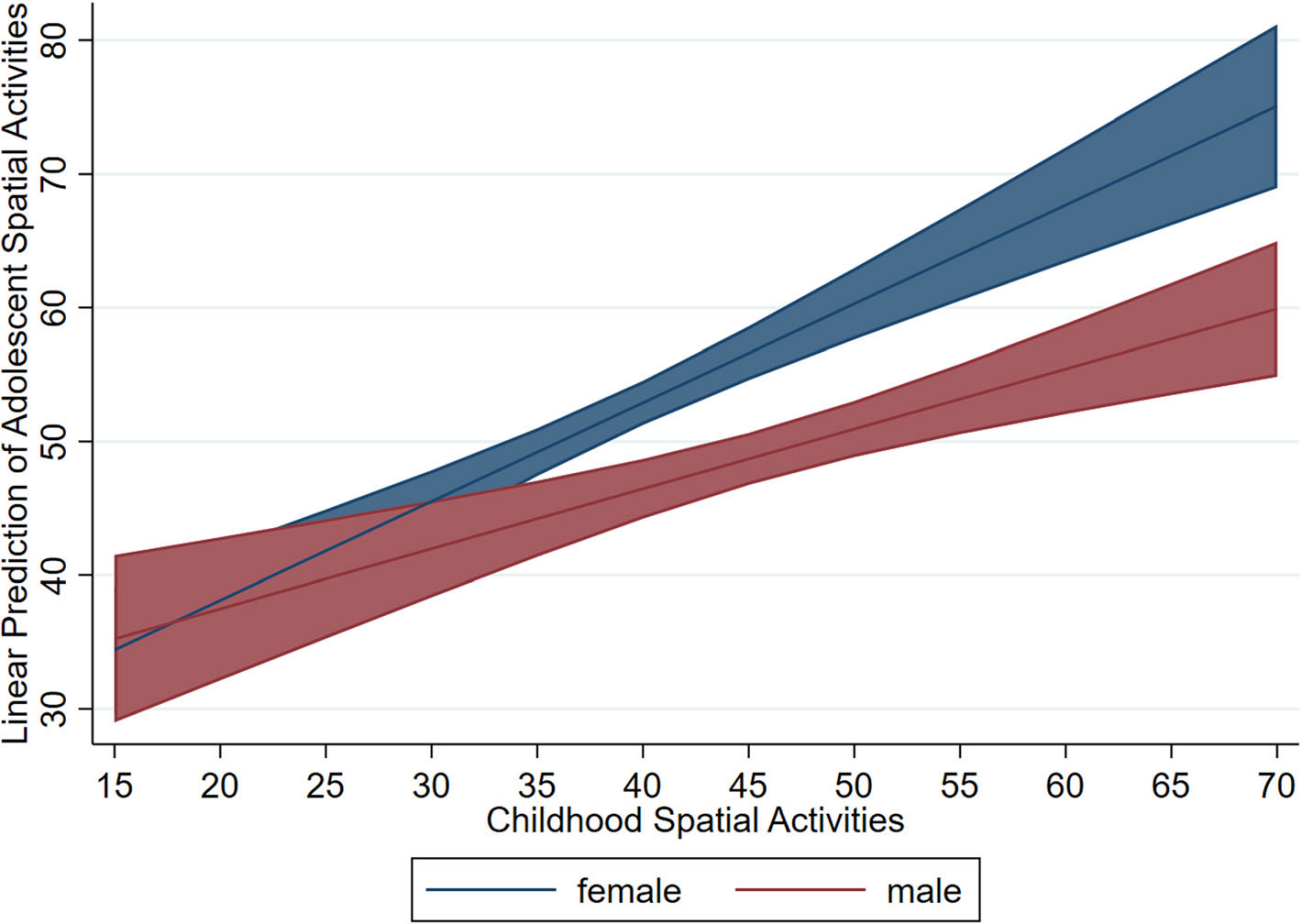
Relation between Childhood Activities and Adolescent Activities by Gender. Predictive margins of gender with 95% CIs

Notably, the model also revealed significant differences by gender, with females scoring higher than males on the SAQ (*b* = − 7.14, *β* = −.28, *SE* = 1.38, *p* < .001). To further assess whether gender influenced the relationship between childhood and adolescent spatial activity participation, we tested a second linear regression model including an interaction between gender and CAQ scores (all additional covariates retained from first model; Table 2). This yielded a significant interaction term (*b* = −.29, *β* = −.55, *SE* = .14, *p* = .04), indicating that the positive association between childhood and adolescent spatial activities was greater for female participants (see Fig. 1 path A). On average, CAQ scores were higher for male participants and male responses were more clustered towards the higher end of the scale (Kolmogorov-Smirnov *p* < 0.001). One interpretation of this interaction is that female participants who scored higher on the CAQ (which was biased towards masculine spatial activities; Table 1) were more strongly engaged in spatial activities relative to same-gender peers than were male participants who reported similar scores. That is, because females scored lower on the CAQ overall, female participants who nonetheless reported greater spatial activity participation during childhood may have been more interested in spatial activities relative to their peers, and thus more likely to continue to engage in spatial activities in adolescence (Fig. 2).

### Spatial activity participation as a predictor of spatial thinking

To address Research Question 2, we conducted multivariate regression analysis (i.e., regression analysis estimating multiple dependent variables) to jointly regress two indicators of spatial thinking (i.e., paper folding and spatial habits of mind) on spatial activities, gender, and relevant covariates. Then, to investigate whether the relations differed by gender—Research Question 3—we added gender as a moderator. Multivariate regression analysis enables estimation of the effect of the predictors on the combination of dependent variables and allows for comparison of the strength of coefficients by outcome measure. That is, it allowed us to test whether the childhood measure of spatial activities predicted each outcome more strongly than adolescent spatial activities (and vice versa). The interpretation of the effects is the same as simple linear regression.

We tested three multivariate regression models (see Table 3). To examine the relations between spatial activities and the outcome measures (Research Question 2) we ran two models. Model 1 included childhood and adolescent activity participation measures. Model 2 included activity participation measures and the interaction between childhood and adolescent activity measures. To investigate whether gender moderated these relations (Research Question 3), in Model 3 we added interactions between gender and activity participation at both timepoints (i.e., CAQ, SAQ). All models included gender, PSAT score, race/ethnicity, and mother’s college completion as covariates.

**Table 3:** Multivariate regression models

	Paper folding					Spatial habits of mind				
	t	B	β	95% CI	*p*	t	B	β	95% CI	*p*
**Model 1**										
CAQ	−0.55	−0.01	− 0.03	[−.07, .04]	0.58	5.34	0.43	0.33	[.27, .59]	**< 0.001**
SAQ	2.57	0.05	0.15	[.01, .09]	**0.01**	1.40	0.08	0.08	[−.03, .20]	0.16
Gender	1.80	0.88	0.10	[−.08, 1.84]	0.07	0.79	1.18	0.05	[−1.78, 4.15]	0.43
Race	−0.57	−0.27	−0.03	[−1.19, .66]	0.57	−1.60	−2.33	−0.09	[−5.18, .53]	0.11
Maternal Education	0.93	0.58	0.05	[−.64, 1.80]	0.35	−1.10	−2.11	−0.06	[−5.88, 1.65]	0.27
PSAT	7.48	0.07	0.41	[.05, .09]	**< 0.001**	2.96	0.08	0.16	[.03, .14]	**0.003**
**Model 2**										
CAQ	0.79	0.07	0.16	[−.10, .23]	0.43	3.10	0.80	0.62	[.29, 1.30]	**0.00**
SAQ	1.68	0.12	0.36	[−.02, .26]	0.10	1.83	0.40	0.39	[−.03, .83]	**0.07**
CAQ X SAQ	−1.01	0.00	−0.33	[.00, .00]	0.31	−1.50	−0.01	−0.50	[−.02, .00]	0.13
Gender	1.77	0.87	0.10	[−.09, 1.83]	**0.08**	0.75	1.14	0.04	[−1.82, 4.10]	0.49
Race	−0.53	−0.25	− 0.03	[−1.17, .68]	0.60	−1.54	−2.24	− 0.08	[−5.09, .61]	0.12
Maternal Education	0.97	0.60	0.05	[−.62, 1.82]	0.33	−1.04	−2.00	−0.06	[−5.75, 1.76]	0.29
PSAT	7.30	0.07	0.40	[.05, .09]	**< 0.001**	2.76	0.08	0.15	[.02, .14]	**0.01**
**Model 3**										
CAQ	0.48	0.02	0.04	[−.06, .09]	0.64	2.18	0.25	0.19	[.02, .47]	**0.03**
SAQ	2.14	0.05	0.15	[.00, .10]	**0.03**	2.11	0.16	0.15	[.01, .30]	**0.04**
Gender	1.76	4.09	0.48	[−.50, 8.68]	**0.08**	−0.74	−5.18	−0.20	[−19.36, 8.81]	0.46
Gender X CAQ	−1.15	−0.06	− 0.34	[−.16, .04]	0.25	2.24	0.36	0.66	[.04, .68]	**0.03**
Gender X SAQ	−0.33	− 0.01	−0.08	[−.10, .07]	0.74	−1.37	−0.18	− 0.34	[−.43, .08]	0.17
Race	−0.71	−0.34	− 0.04	[−1.27, .59]	0.48	−1.57	−2.26	− 0.09	[−5.15, .58]	0.12
Maternal Education	1.03	0.64	0.06	[−.58, 1.86]	0.30	−1.28	−2.43	−0.07	[−6.20, 1.31]	0.20
PSAT	7.26	0.07	0.40	[.05, .09]	**< 0.001**	2.99	0.09	0.17	[.03, .14]	**0.003**

*SAQ* Adolescent spatial activities questionnaire, *CAQ* Childhood activities questionnaire – spatial subscale

*N* = 305

When childhood and adolescent activities were included in a model predicting paper folding (Model 1), the model accounted for a significant portion of the variance (F_6,298_ = 12.95, *p* < .001, *R*^*2*^ = .21). Adolescent activities were a significant predictor (*b* = .05, *β =* .15, *SE =* .02, *p* = .01); however, childhood activities were not (*b* = −.01, *β =* −.03, *SE =* .03, *p* = .58). The interaction between childhood activities and adolescent activities (Model 2) was non-significant, and this interaction term was dropped in the subsequent model. Model 3 included gender x childhood activities and gender x adolescent activities interaction terms. Although the overall model was significant (F_8,296_ = 9.99, *p* < .001, *R*^*2*^ = .21), neither gender X activity interaction term was significant.

**Fig. 3:**
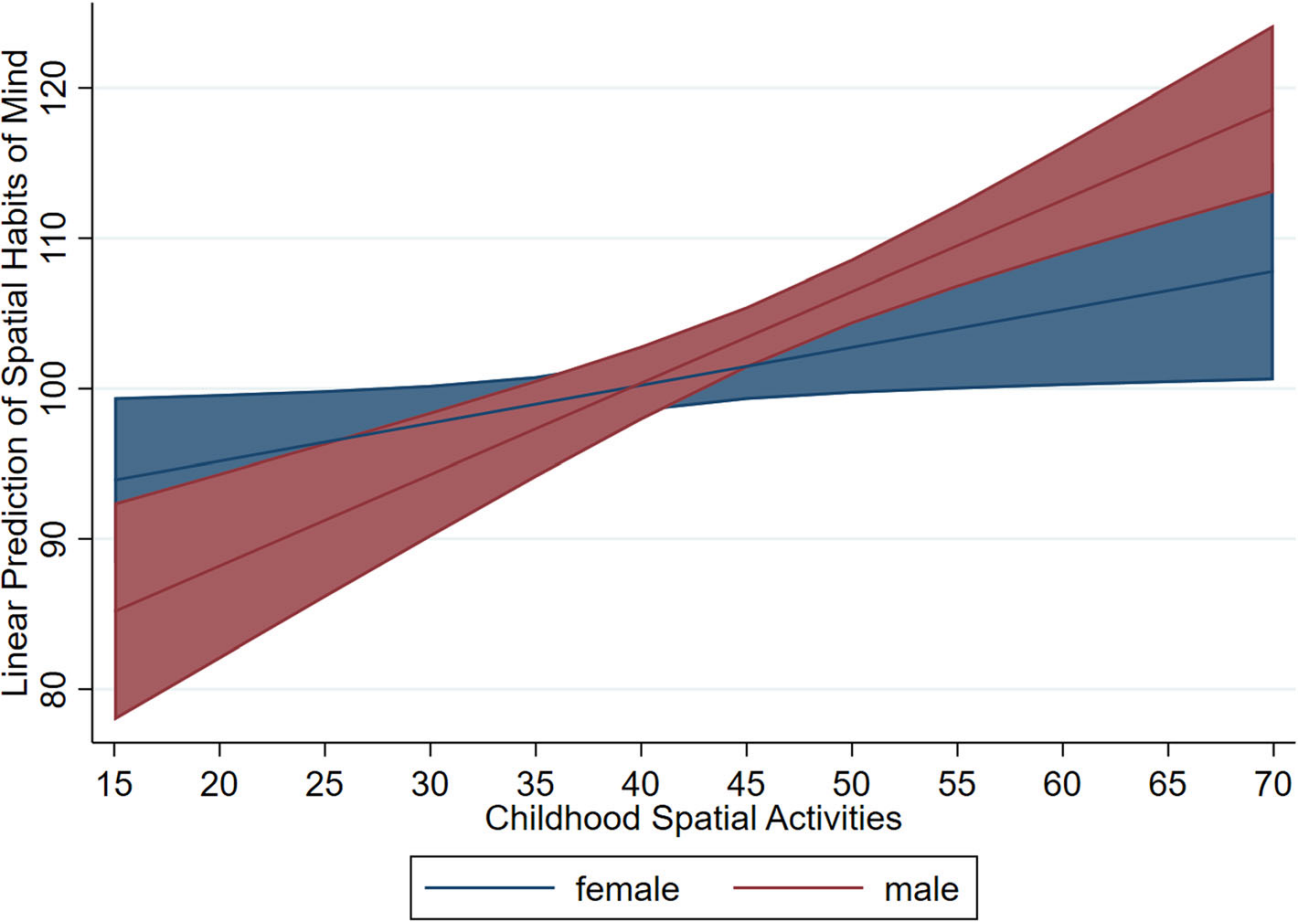
Relation between Childhood Activities and Spatial Habits of Mind by Gender. Predictive margins of gender with 95% CIs

For spatial habits of mind, including spatial activities in childhood and adolescence as predictors (Model 1; F_6,298_ = 11.67, *p* < .001, *R*^*2*^ = .19) revealed that spatial activities in childhood were a significant predictor (*b* = .43, *β* = .33, *SE* = .08, *p* < .001), while adolescent spatial activities were not (*b* = .08, *β =* .08, *SE =* .06, *p* = .16). Model 2 did not reveal a significant interaction between childhood activities and adolescent activities, so the interaction was dropped from the subsequent model. When including gender X activity interactions (Model 3), the overall model was significant (F_8,296_ = 11.61, *p* < .001, *R*^*2*^ = .20). With these interactions included, there were significant main effects of both childhood activities (*b* = .25, *β* = .19, *SE* = .11, *p* = .03) and adolescent activities (*b* = .16, *β* = .15, *SE* = .07, *p* = .04). This was in contrast to Model 1 (no interaction terms), which did not find a significant effect of adolescent activities. Although there was no main effect of gender, gender significantly moderated the effect of childhood activities on spatial habits of mind (*b* = .36, *β* = .66, *SE* = .16, *p* = .03). Specifically, the relation between childhood activities and spatial habits of mind was stronger for males than for females (see Fig. 3). There was no significant interaction between adolescent spatial activities and gender.

One benefit of conducting multivariate regression rather than separate linear regressions for each outcome variable is that we can compare the strength of coefficients on each outcome. Using Model 3, we examined two comparisons: (a) the relation between childhood activities and each outcome measure, and (b) the relation between adolescent activities and each outcome measure. There was a significant difference in the coefficients for childhood activities, indicating that childhood activities were more predictive of spatial habits of mind than of paper folding (F_1, 296_ = 4.12, *p* = .04). However, the coefficients for adolescent spatial activities were not statistically different between paper folding and spatial habits of mind (F_1, 296_ = 2.00, *p* = .16). This suggests that the extent to which adolescent activities predicted paper folding was not greater than its prediction of spatial habits of mind.

## Discussion

In the present study, we investigated participation in spatial activities during childhood and adolescence, and examined the extent to which activity participation predicted adolescents’ spatial thinking skills and strategies. Prior research has recognized that participation in spatial activities is positively related to spatial thinking. The present study extends previous research by examining the stability of spatial activity participation during two critical developmental stages, and the unique associations between spatial activities during these developmental periods with spatial skills and spatial habits of mind. Further, we examined whether gender moderated these relations.

### Spatial activity participation remains stable over time

Results of the present study indicated that students who participated in more spatial activities during childhood reported greater participation in spatial activities during high school, even when controlling for gender and other demographic covariates. Notably, there was an interaction with gender: the association between childhood spatial activities and adolescence spatial activities was stronger for females relative to males. This suggests that while involvement in spatial activities remained relatively stable between childhood and adolescence it was even more consistent for females. It may be that participating more frequently in spatial activities in childhood sets females on a path to more frequent spatial activity participation in adolescence. Although future research will be necessary to directly assess whether interventions promoting spatial activities in childhood increase later activity participation, the results of the present study indicate that spatial activity participation among young girls may be particularly important to consider in future research.

### Spatial activity participation predicts spatial thinking skills and strategies

We found that spatial activity participation predicted different facets of adolescents’ spatial thinking depending on: (a) whether the activities took place in childhood or adolescence (see Fig. 1b paths B and C) and (b) whether the outcome measure was spatial skills or spatial strategies. There was some variation in patterns of statistical significance depending on whether gender was included as a moderator. In all models, childhood spatial activities were related to spatial habits of mind, whereas adolescent spatial activities were related to spatial skills. The possibility that there is a multiplicative effect of participating in spatial activities during both childhood and adolescence was not supported in the current study. When gender was included as a moderator, adolescent spatial activities also predicted spatial habits of mind. Even though childhood activities predicted adolescent activities and adolescent activities predicted spatial skills, childhood activities was indirectly rather than directly related to spatial skills. When accounting for gender differences in the relationship between activity participation and spatial thinking (i.e., gender X activity interactions), adolescent spatial activities were also predictive of spatial habits of mind. The relation between adolescent activities and spatial skills was similar in strength to the relation between adolescent activities and spatial habits of mind. It is also notable that, even when accounting for adolescent activities, participants who reported engaging in more childhood spatial activities also tended to think about problems in a more spatial way (i.e., spatial habits of mind). This extends prior research studies that have focused on the relationship between spatial activities and spatial skills. The relation between childhood spatial activities and spatial habits of mind was even stronger for males than females, further emphasizing the need to understand spatial activity participation in young girls.

### Gender moderates the relations between spatial activities and spatial thinking

The role of gender on the effect of spatial activities was of interest given conflicting findings in prior research. Results indicated that the relation between childhood spatial activities and spatial habits of mind (see Fig. 1b path B) was stronger for males than for females. This aligns with previous findings that gender moderates the relation between spatial activities and spatial thinking, and supports prior research suggesting that the relation is stronger for males than females (Connor & Serbin, 1977; González-Calero et al., In press; Wong & Yeung, 2019). As hypothesized by others (Reich et al., 2018; Wong & Yeung, 2019), it may be that boys engage in spatial activities in ways that better supports spatial habits of mind. However, gender did not moderate the relations between spatial activities and spatial skills, suggesting that spatial activities are related to spatial skills regardless of gender (Jansen et al., 2018; Moreau et al., 2012; Vander Heyden et al., 2017).

### Implications for future research and practice

#### Understanding spatial activity participation across development

The present study underscores the importance of accounting for spatial activity participation during multiple developmental stages, and highlights the need for future research to account for how spatial activity participation unfolds over time. Although prior research has documented that spatial activities are important (Fig. 1a; e.g., Nazareth et al., 2013), the consistency of spatial activity preferences over time (Fig. 1b) has been unaddressed. In this study, we were interested in general proclivities to engage in spatial activities. We examined overall tendencies to participate in spatial activities rather than examine the effect of specific types of activities (e.g., sports vs. toys, masculine activities vs. feminine activities). The specific items were representative activities and reflected differences in common activities in childhood versus adolescence. Given differences in the content of the measures (see Appendix), it is possible that the strength of the relations may be a function of their degree of overlap. Accordingly, we cannot account for whether specific types of spatial activities are more or less stable over time, or whether certain activities incur more benefit for spatial thinking. Relatedly, it is possible that the observed influence of gender on spatial activity stability may have stemmed from the emphasis on masculine spatial activities in childhood questionnaires. For instance, the Childhood Activities Questionnaire (CAQ) used in the present study was comprised of predominantly masculine spatial activities, whereas the Adolescent Spatial Activities Questionnaire (SAQ) consisted of masculine, feminine, and neutral spatial activities. Indeed, male participants reported higher childhood spatial activity participation, although there did not appear to be a ceiling effect. Therefore, this may have decreased the strength of the correlation between childhood and adolescent activities for males. These gender differences were not found for the SAQ, which contained a wider-range of activities.

Adolescents in this study provided retrospective reports of spatial activity participation in adolescence (since starting high school) and childhood (before high school). Limitations of self-report (e.g., poor memory, false perceptions of self) are important to acknowledge. It may be possible that adolescents in the present study recalled previous experience with activities based on gendered schemas (e.g., boys recall playing with more masculine objects in childhood and adolescence). However, the findings in the current study held even when controlling for the effects of gender. Therefore, it is unlikely that students relied solely on gendered expectations in their self-reports. Some researchers have used parental report (e.g., Robert & Héroux, 2004). However, many limitations of self-report, such as memory challenges and recall based on gendered schemas, also apply to retrospective reports from others such as parents, and the reports from parents of adolescents are typically related to adolescent self-report (e.g., Barr-Anderson, Robinson-O’Brien, Haines, Hannan, & Neumark-Sztainer, 2010). Although it would have been ideal to corroborate self-report with parental report, the practical feasibility of obtaining parental surveys of adolescents limited our ability to collect these data in the present study.

#### Expanding conceptualizations of spatial thinking in studies of spatial activities

When developing theoretical models and educational interventions, it is worth considering that spatial activities may be related to multiple facets of spatial thinking. Prior research has predominately focused on the relation between spatial activities and spatial skills (see Moè et al., 2018 for an exception); however, spatial skills are only one aspect of developing students as spatial thinkers (Kim & Bednarz, 2013; NRC, 2006). Indeed, present study suggests that childhood spatial activities may be even more important for spatial strategies than for spatial skills. However, even spatial skills are not unidimensional (Uttal et al., 2013). Paper folding, the assessment of spatial skills used in the present study represents just one facet of spatial skills: mental rotation or “folding” (Harris, Newcombe, & Hirsh-Pasek, 2013). This is distinct, however, from other spatial skills such as visual search (e.g., Karp & Konstadt, 1963) or navigation (e.g., Nazareth, Newcombe, Shipley, Velazquez, & Weisberg, 2019). We selected paper folding for several reasons. First, due to our interest in better understanding gender differences, we selected paper folding as a measure of spatial skills that has a history of reported gender differences in performance (Blazhenkova & Kozhevnikov, 2010; Ramful & Lowrie, 2015). Second, given testing constraints, it was necessary to select a measure that could be given in a short amount of time to a group of students without requiring computers. Further, in order to use propensity score methods to identify participants to be invited to participate in a further study of spatial skills, we selected paper folding as a measure of spatial skills that was likely to be correlated with the central measures to be used in the follow-up study (e.g., Mental Rotation Test; Blajenkova, Kozhevnikov, & Motes, 2006; Hegarty & Waller, 2004). Whether spatial activity participation during childhood and/or adolescence is differentially predictive of different types of spatial skills remains an open question. Future research should examine multiple different measures of spatial skills in relation to spatial activities, in order to better capture the multidimensional nature of spatial skills.

Finally, future studies should examine the directionality of the relationship between spatial activities and spatial thinking. This includes investigating whether spatial activities predict spatial skills, or whether children with better spatial skills are more inclined (or encouraged by others) to partake in spatial activities. As noted in Fig. 1b, the present study examined some of the relations between spatial activities and spatial thinking between childhood and adolescence. However, given the absence of a measure of childhood spatial skills in this study, and the cross-sectional nature of the study design, it was not possible to explore all of the possible relations. Future research using longitudinal designs, particularly those utilizing interventions, would provide a clearer, causal mechanism for the relations between spatial activity participation and spatial thinking skills and strategies.

## Conclusions

Participation in activities that support spatial thinking, ranging from play with blocks and cars to participation in sports and dance, often take place in out-of-school and recreational contexts. Yet, these activities have the potential to support the cognitive skills and ways of thinking that foster students’ learning, especially in STEM (Stieff, Dixon, Ryu, Kumi, & Hegarty, 2014; Verdine, Michnick Golinkoff, Hirsh-Pasek, & Newcombe, 2014; Wai et al., 2009). In addition to considering whether spatial activities are important, it is worth considering when and for whom these types of activities provide the most benefit, and for what types of spatial thinking outcomes spatial activities are most relevant.

## Data Availability

The datasets and materials analyzed in the current study are available from the corresponding author on reasonable request.

## Appendix

### Spatial activities included in the activities questionnaire measures

**Childhood Activities Questionnaire** (Spatial Subscale) from Cherney and Voyer (2010).

Air hockey; Alphabet blocks or similar; Baseball; Basketball; Cars and trucks; Construction blocks (other than Legos); Dodge ball; Football; Lego blocks; Ping pong; Play musical instruments; Puzzles; Shooting pool/billiards.

**Spatial Activities Questionnaire Short Form** from Signorella et al. (1986).

- ***Gender neutral activities***: Tennis (serve and volley; place shots); Racquetball; Bowling; Diving; Dodgeball; Drawing (in 3-D perspective); Painting (in 3-D perspective); Pingpong (table tennis); Softball; Volleyball
- ***Masculine activities***: Baseball; Building go-carts or soapbox cars; Building model trains or racing car sets; Car repair; Carpentry; Electrical repair; Making or fixing radios/stereos; Tackle football; Target shooting (pistol or rifle); Touch football
- ***Feminine activities***: Ballet (with pirouettes, turns requiring spotting); Baton twirling (with tossing in air); Crochet (pieces needing seams); Embroidery/needlepoint (not following printed pattern); Figure skating; Gymnastics; Interior decorating; Knitting multi-color patterns; Making patchwork quilts; Sketching clothes design
